# Platelet-independent adhesion of calcium-loaded erythrocytes to von Willebrand factor

**DOI:** 10.1371/journal.pone.0173077

**Published:** 2017-03-01

**Authors:** Michel W. J. Smeets, Ruben Bierings, Henriet Meems, Frederik P. J. Mul, Dirk Geerts, Alexander P. J. Vlaar, Jan Voorberg, Peter L. Hordijk

**Affiliations:** 1 Department of Molecular Cell Biology, Sanquin-Academic Medical Center Landsteiner Laboratory, Amsterdam, The Netherlands; 2 Department of Plasma Proteins, Sanquin-Academic Medical Center Landsteiner Laboratory, Amsterdam, The Netherlands; 3 Department of Pediatric Oncology/Hematology, Erasmus University Medical Center, Rotterdam, The Netherlands; 4 Department of Intensive Care Medicine, Amsterdam Medical Center, Amsterdam, The Netherlands; 5 Department of Physiology, VU University Medical Center, Amsterdam, The Netherlands; Ludwig-Maximilians-Universitat Munchen, GERMANY

## Abstract

Adhesion of erythrocytes to endothelial cells lining the vascular wall can cause vaso-occlusive events that impair blood flow which in turn may result in ischemia and tissue damage. Adhesion of erythrocytes to vascular endothelial cells has been described in multiple hemolytic disorders, especially in sickle cell disease, but the adhesion of normal erythrocytes to endothelial cells has hardly been described. It was shown that calcium-loaded erythrocytes can adhere to endothelial cells. Because sickle erythrocyte adhesion to ECs can be enhanced by ultra-large von Willebrand factor multimers, we investigated whether calcium loading of erythrocytes could promote binding to endothelial cells via ultra-large von Willebrand factor multimers. We used (immunofluorescent) live-cell imaging of washed erythrocytes perfused over primary endothelial cells at venular flow rate. Using this approach, we show that calcium-loaded erythrocytes strongly adhere to histamine-stimulated primary human endothelial cells. This adhesion is mediated by ultra-large von Willebrand factor multimers. Von Willebrand factor knockdown or ADAMTS13 cleavage abolished the binding of erythrocytes to activated endothelial cells under flow. Platelet depletion did not interfere with erythrocyte binding to von Willebrand factor. Our results reveal platelet-independent adhesion of calcium-loaded erythrocytes to endothelium-derived von Willebrand factor. Erythrocyte adhesion to von Willebrand factor may be particularly relevant for venous thrombosis, which is characterized by the formation of erythrocyte-rich thrombi.

## Introduction

Healthy erythrocytes do not bind to the endothelial cells (ECs) that line the vascular wall. In contrast, in multiple hematologic disorders [1–4] and most prominently in sickle (SS) cell disease, erythrocyte adhesion to ECs does occur [5]. This causes vaso-occlusive events that impair blood flow which, in turn, can result in ischemia and tissue damage [5]. Since Hebbel *et al*. observed the binding of SS erythrocytes to endothelial cells [6, 7], many mechanisms that may cause erythrocyte-EC adhesion have been described [5]. One of the most studied mechanisms is the adhesion of SS erythrocytes to ECs via ultra-large VWF (ULVWF) multimers [8, 9]. *In vitro* EC-derived ULVWF multimers can greatly enhance the adherence of SS erythrocytes to ECs, but only slightly augment the adhesion of normal erythrocytes [8, 10]. An *ex vivo* rat mesocecum perfusion model confirmed these results and showed that the release of VWF from desmopressin-stimulated ECs significantly increased adhesion of SS erythrocytes to the venular endothelium [9]. Also a correlation between the clinical severity of sickle cell disease, deduced from the extent of hemolysis, and plasma levels of total active VWF was found [11].

Although the SS erythrocyte-EC interaction via ULVWF is well-accepted, the adhesion of normal erythrocytes to ECs has hardly been described. In addition to SS erythrocytes, also calcium-loaded erythrocytes can adhere to ECs [7]. Furthermore, in the *ex vivo* rat mesocecum model perfused with desmopressin, it was shown that ULVWF released from ECs also promoted the adhesion of normal erythrocytes to the venular endothelium [12]. Based on these previous findings, we investigated whether calcium loading of erythrocytes could enhance the binding of erythrocytes to ECs via ULVWF multimers. Our results reveal platelet-independent adhesion of calcium-loaded erythrocytes to endothelium-derived VWF.

## Methods

### Erythrocytes and platelets isolation

Blood studies were approved by the Sanquin Research Institutional Medical Ethical Committee in accordance with the Dutch regulations and the 1964 Declaration of Helsinki standards. Whole blood was collected in 3.8% sodium citrate tubes (Greiner Bio-One) from healthy, anonymized volunteers that provided written informed consents which were approved by the Sanquin Research Institutional Medical Ethical Committee. Platelet-rich plasma (PRP) was separated from the erythrocyte-rich pellet by centrifugation at 200x*g* (15 minutes). The erythrocytes were washed in SAGM (150 mM NaCl, 1.25 mM adenine, 28.82 mM mannitol, 49.95 mM D-glucose) and resuspended in SAGM (concentration ~ 3.5x10^9^ cells/ml).

Acid citrate dextrose was added to the PRP (10% v/v) prior to centrifugation at 2000x*g* (5 minutes). The platelet pellet was washed twice with wash buffer (36 mM citric acid, 103 mM NaCl, 5 mM KCl, 5 mM EDTA (ethylenediaminetetraacetic acid), 56 mM D-glucose, pH 6.5 containing 0.35% [wt/vol] bovine serum albumin) and resuspended to 200-400x10^6^ cells/ml in HEPES(+) buffer (132 mM NaCl, 20 mM HEPES (N-2-hydroxyethylpiperazine-N'-2-ethanesulfonic acid), 6 mM KCl, 1 mM MgSO_4_•7H_2_O, 1.2 mM K_2_HPO_4_•3H_2_O, 2.5 mM CaCl_2_, 5.5 mM D-glucose, pH 7.4).

Cells were counted using an Advia 2120 Hematology System hematology analyzer (Siemens).

### Erythrocyte calcium loading or depletion

Erythrocytes (10^8^ cells/ml) suspended in HEPES(+) (2.5 mM calcium) buffer, HEPES buffer with the indicated concentrations of calcium, or HEPES buffer supplemented with 5 mM EGTA (ethylene glycol tetraacetic acid) were treated with 1 μM ionomycin for 1 hour. Treated erythrocytes were washed in HEPES(+) buffer with the equivalent calcium concentration, stored at room temperature and resuspended, prior to a flow assay, in 1% BSA/HEPES(+) or 1% BSA/HEPES buffer without calcium supplemented with 5 mM EGTA. Both buffers were supplemented with 100 μM histamine.

### EC culture and flow chambers

Pooled human umbilical vein ECs (HUVECs) (Lonza), human aortic ECs (HAECs) (Lonza), human microvascular ECs (HMEC-1) [13] (obtained from Dr Ades [Centers for Disease Control and prevention, Atlanta, GA]) and blood outgrowth ECs (BOECs) (isolated as described previously [14]) were cultured in fibronectin (FN)-coated flasks in EBM-2 medium (Lonza) supplemented with EGM-2 SingleQuot Kit Suppl. & Growth Factors (Lonza). ECs (7.5x10^4^ cells/ml) were seeded in FN-coated μ-Slide VI 0.4 ibiTreat flow chambers (Ibidi). The ECs were cultured for 5 days before flow experiments. When indicated, the ECs were stimulated with 10 ng/ml TNF-α (Peprotech) for 24 hours. HUVECs were used until passage 5, BOECs and HAECs until passage 7, and the cell line HMEC-1 until passage 15.

### Live imaging of erythrocyte binding to ECs under flow

Erythrocytes (10^8^ cells/ml) were incubated for one hour with 1 μM ionomycin or 10 μM valinomycin. DMSO or H_2_O were used as controls. Erythrocytes (10^8^ cells/ml) with or without platelets (concentrations as indicated) or with or without monoclonal anti-VWF CLB-RAg20-coupled [15] fluoresbrite YG microspheres (∅ 3 μm) (Polysciences) supplemented with 100 μM histamine, 1 U/ml thrombin, or vehicle were perfused for 10 minutes over ECs. All perfusions were performed with a syringe pump (NE-1010 ProSense) at 0,57 ml/min which gives a wall shear stress of 0.72 dyne/cm^2^ and a wall shear rate of 100 s^-1^. Real-time imaging was performed by an Axiovert 200M microscope (Carl Zeiss Microscopy) with an Orca-R^2^ camera (Hamamatsu) using a 20x air objective.

### Live fluorescent imaging under flow

Ionomycin- or control-treated erythrocytes (10^9^ cells) were stained using PKH67GL (Sigma Aldrich). Platelets (4x10^7^ cells) were stained using anti-CD42b-APC (Life Technologies). Alexa Fluor^®^568-labelled anti-VWF (Dako) antibody was prepared using a Zenon^®^ Alexa Fluor^®^ 568 Rabbit IgG Labeling Kit (Molecular Probes). Erythrocytes (10^8^ cells/ml) with or without platelets (4x10^6^ cells/ml), anti-VWF Alexa Fluor^®^568 (1.2 μg/ml) and 100 μM histamine were perfused over ECs for 10 minutes. Real-time imaging was performed using the Axiovert 200M with a 40x oil objective (NA 1.3).

### Effects of ADAMTS13 on erythrocyte binding to ECs under flow

Ionomycin-treated erythrocytes (10^8^ cells/ml) were perfused with 100 μM histamine over ECs for 10 minutes. Without flow interruption, perfusion was continued with or without 5% autologous platelet-poor-plasma or 5 μg/ml recombinant ADAMTS13 (purified as described before [16]) for 10 minutes. At each time point images were acquired using an Axiovert 200M microscope with a 20x air objective.

### VWF knockdown

Six pre-tested VWF-specific shRNA constructs (Sigma MISSION^®^ TRC-Hs 2.0 shRNA library) [17] were used in VWF knockdown and function interference experiments. The results shown were obtained with TRCN0000373946 (ACATGGAAGTCAACGTTTATG, targeting nt 6360–6380 of VWF RefSeq NM_000552.3). The non-targeting hairpin control SHC002 (Sigma Aldrich) was used as a negative control. shRNA constructs were packaged into lentiviral particles in HEK293T cells after TransIT^®^LT1 (Mirus) mediated transfection together with the pHDM-HgPM2, pRC-CMV-Rev1b, pHDMG-G, and pHDM-TAT1B packaging plasmids for 24 and 48 hours. HUVECs were transduced twice with a 24 hour interval. Forty eight hours after the first transduction, 7.5x10^4^ EC/ml were seeded in flow chambers and cultured for 6 days, used for flow assays, fixed using paraformaldehyde (PFA) (4% w/v) for 15 minutes, or lysed in sample buffer (0.125 M UltraPure Tris-HCl (tris(hydroxymethyl)aminomethane), 10% glycerol, 2% SDS, 2% β-mercapto-ethanol, 0.001% bromophenol blue, pH 6.8).

### FACS analysis

Erythrocytes (10^8^ cells/ml) in HEPES(+) buffer or HEPES buffer with 5 mM EGTA were treated with 1 μM ionomycin or control (DMSO) for 1 hour. Erythrocytes (10^5^ cells) were incubated with BSA (1% w/v) for 15 minutes and subsequently stained using Annexin V-FITC (BD Pharmingen) and anti-CD235a-PE (Sanquin) for 15 minutes. Phosphatidylserine (PS) exposure was determined with a FACSCanto II flow cytometer (BD). Data were analyzed using FlowJo version 10.0.8r1 (LLC).

### Erythrocyte sorting

Erythrocyte ultra-purification was performed by fluorescence-activated cell sorting (FACS) using a FACSAria III cell sorter (BD). Erythrocyte concentrates (10^7^ cells/ml) were stained using anti-CD235a-FITC (Sanquin) and anti-CD42b-APC (Life Technologies) for 30 minutes. Erythrocytes were purified by positive sorting for CD235a and negative sorting for CD42b with doublet exclusion by using FSC-H and FSC-W.

### Western blot

Cell lysates were run on a 12.5% polyacrylamide gel and transferred onto Amersham^™^ Protan^™^ 0.2 μm nitrocellulose blotting membranes (GE Healthcare). Membranes were stained using anti-ICAM1 (Santa Cruz Biotechnology), anti-actin (Sigma Aldrich), anti-VWF (Dako) overnight at 4°C and counterstained with anti-mouse HRP (Dako) or anti-rabbit HRP (Dako) for 1 hour. Immobilized antigens were detected by chemiluminescence using Pierce^®^ECL Western Blotting Substrate (Thermo Scientific) and exposed on Fuji Medical X-Ray films (Fujifilm).

### Immunofluorescence imaging

Paraformaldehyde-fixed samples were permeabilized with Triton X-100 (0.5% v/v) for 5 minutes and blocked with BSA (2% w/v) for 30 minutes. ECs were stained with anti-VWF (Dako), anti-VWF CLB-RAg20 (Sanquin), anti-VE-cadherin (Santa Cruz Biotechnology), or anti-PECAM (Sanquin) for 1 hour. Next, ECs were stained with anti-Rabbit Alexa Fluor^®^488 (Thermo Fisher) and anti-Goat Alexa Fluor^®^633 (Thermo Fisher) for 1 hour. Fluorescent images were acquired using a LSM510 Meta confocal microscope (Carl Zeiss) with a EC Plan-Neofluar 40x oil objective (NA 1.3).

### VWF ELISA

ECs were stimulated with HEPES(+) buffer containing 100 μM histamine for 10 minutes or lysed in HEPES(+) buffer with Triton X-100 (1.0% v/v). Intracellular or secreted VWF was assayed by sandwich enzyme-linked immunosorbent assay (ELISA) as described previously [18] using rabbit polyclonal anti-VWF (DAKO) as coating antibodies and HRP-conjugated rabbit polyclonal anti-VWF (DAKO) for detection of bound VWF. Standard curves were made from dilutions of culture supernatants of HEK293 cells stably producing recombinant VWF. Optical density levels measured below 2 times the blank were set to 0 pM.

### Imaging and analysis

Images were taken using Zen 2012 software version 1.1.2.0 (Carl Zeiss). For all image processing ImageJ version 1.49g (https://imagej.nih.gov/ij/) was used. Weibel-Palade bodies were quantified using the functions “threshold” and “analyze particles” within regions of interest based on the VE-cadherin staining. Erythrocyte adhesion was quantified using the ImageJ plugin Cell Counter. Erythrocytes were qualified as firmly adherent when they remained immobile for 5 seconds before and 5 seconds after each time point otherwise they were qualified as loosely adherent. For each experiment, tile scans (3800 μm width) were made (S1 Fig). Each data point represents the average number of adherent erythrocytes within 15 fields of view (FOV; 1 FOV = 419.23x319.41 μm) (S1 Fig).

### Statistical analysis

Data shown are mean ±SD. Statistical comparisons were made using an unpaired, 2-tailed Student's t test, 1-way ANOVA followed by Bonferroni's post-hoc test, or 2-way ANOVA followed by Bonferroni's post-hoc test, where appropriate. Statistical analysis was performed using GraphPad Prism version 6.04 (GraphPad Software). For all tests, results were considered statistically significant at P < 0.05. Graphs showing individual data points are provided in S8 and S9 Figs.

## Results

### Erythrocyte binding to ECs

**Fig 1:**
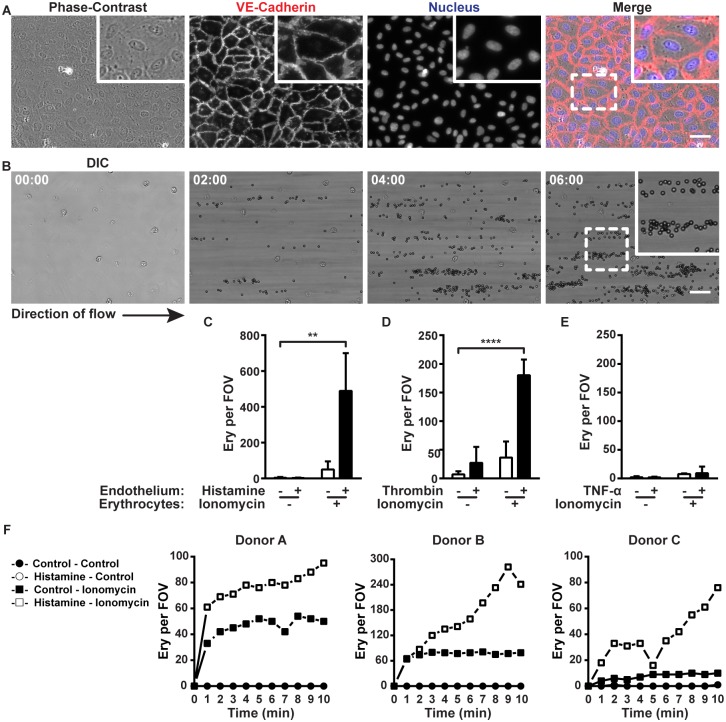
Erythrocyte binding to ECs. (A) HUVECs cultured in perfusion chambers and stained for VE-Cadherin (red) and nuclei (blue). The dashed box corresponds to the zoomed region. Scale bar represents 50 μm. (B) Erythrocytes treated for 1 hour with ionomycin (1 μM) were perfused over histamine- (100 μM) activated HUVECs (37°C and 5% CO_2_). Adhesion was recorded for 10 minutes. Images are stills from the video in S1 Video at the indicated times in minutes. The arrow indicates the direction of the flow. The dashed box corresponds to the zoomed region. Scale bar represents 50 μm. (C) Quantification of adhesion of ionomycin- (1 μM) or control-treated (1 hour) erythrocytes perfused (10 minutes) with or without histamine (100 μM) over HUVECs. A schematic outline of the quantification method is shown in S1 Fig. (D) Ionomycin- or control-treated erythrocytes perfused with or without thrombin (1 U/mL) over HUVECs. (E) Ionomycin- or control-treated erythrocytes perfused over TNF-α- (10 ng/ml) or control-treated (24 hours) HUVECs. (F) Kinetics of ionomycin- or control-treated erythrocyte accumulation after perfusion with or without histamine (100 μM) over HUVECs (Control-Control: ●; Histamine-Control: ○; Control-Ionomycin: ■; Histamine-Ionomcyin □). Adhesion of erythrocytes from 3 independent donors was quantified at a 1 minute time interval for 10 minutes. For C-E the average number of adherent erythrocytes within 15 fields of view (FOV; 1 FOV = 419.23x319.41 μm) was used. For F the absolute number of adherent erythrocytes within 1 field of view was used. Values are means ± SD (n = 3; Each n represents single independent experiments using cells from different donors). Statistical analysis was performed using a 2-way ANOVA in which all means were compared with the double-blank control followed by Bonferroni's post-hoc test. ** *P* < 0.01; **** *P* < 0.0001.

To analyze the adhesion of normal erythrocytes to primary human ECs, we used *in vitro* flow chambers in which washed erythrocytes were perfused over cultured EC monolayers at a wall shear rate of 100 s^-1^ and wall shear stress of 0,72 dyne/cm^2^ corresponding to the conditions in post-capillary venules [19] or veins [20]. First we confirmed that the ECs formed a confluent and stable monolayer within the perfusion chambers by staining for VE-cadherin. (Fig 1A). We also confirmed that the ECs kept their endothelial phenotype after culturing in the perfusion chambers by staining for VWF and VE-cadherin (S2 Fig) or PECAM (S2 Fig).

In contrast to SS erythrocytes, healthy erythrocytes do not bind to ECs [7], which we confirmed by perfusing freshly isolated normal erythrocytes over EC monolayers. An example of this experiment can be seen in S1 Video. Previously, the release of ULVWF multimers from ECs was shown to cause adhesion of normal erythrocytes to the venular endothelium in an *ex vivo* rat mesocecum model [12]. Thrombin and histamine may induce increased release of endothelial ULVWF multimers during episodes of vascular injury, inflammation, and infection [21, 22]. Yet, in our flow-model, washed erythrocytes that contained virtually no platelets and white blood cells as shown in Table 1, showed little adhesion to ECs activated with 100 μM histamine or 1 U/ml thrombin (Fig 1C and 1D).

**Table 1:** Blood cell counts measured from the washed erythrocyte concentrates used in Fig 1C and 1D.

	Mean absolute number (x10^9^ cells / L) and range (x10^9^ cells / L)	Percentage of total and range (%)
**Erythrocytes**	3267 (2450–3700)	99.88 (99.75–99.97)
**Platelets**	2.67 (0–6)	0.10 (0.00–0.24)
**White blood cells**	0.82 (0.16–1.34)	0.02 (0.007–0.036)

However, since it has been described that calcium-loaded erythrocytes can adhere to ECs [7], we tested the effect of calcium loading on erythrocyte-EC interaction. Erythrocyte treatment with 1 μM ionomycin induced a rapid calcium influx followed by phosphatidylserine exposure and cell volume decrease (S2 Fig). Interestingly, calcium-loaded erythrocytes adhered to non-treated ECs (Fig 1C and 1D and S1 Video). Moreover, a double hit experimental set-up in which erythrocytes were loaded with calcium and perfused over histamine- or thrombin-activated ECs, induced strong and abundant adhesion of erythrocytes to ECs (Fig 1C and 1D and S1 Video). In contrast, the pro-inflammatory cytokine TNF-α, which is also secreted during injury, inflammation and infection, did not promote the binding of normal or calcium-loaded erythrocytes to ECs, despite the upregulation of EC adhesion molecules such as intercellular adhesion molecule 1 (Fig 1E and S2 Fig). To test the effect of a different ionophore on erythrocyte adhesion to ECs we also stimulated erythrocytes with the potassium ionophore valinomycin. Although valinomycin caused a significant potassium efflux from erythrocytes, only minor adhesion of treated erythrocytes to ECs could be observed (S3 Fig). To investigate the dynamics of the erythrocyte-endothelium adhesion process, ionomycin- or control-treated erythrocytes from 3 independent donors were perfused with or without histamine over ECs. Control treated erythrocytes showed both in the presence and absence of histamine no adhesion to ECs (Fig 1F). On the contrary, the adhesion of calcium-loaded erythrocytes in the absence of histamine increased during the first minutes after which an equilibrium between attaching and detaching cells established (Fig 1F). The double hit experimental set-up induced strong and abundant adhesion of erythrocytes to ECs within the first minutes followed by a gradual increase in absolute numbers of adherent cells (Fig 1F).

We also looked at the ratio between firmly adherent erythrocytes and loosely adherent erythrocytes (S4 Fig). Although some erythrocytes detach after adhering to ECs (S1 Video), quantification showed that both in the ionomycin-treated condition as in the double hit experimental set-up the absolute number of loosely adherent erythrocytes is small (S4 Fig). The maximal amount of loosely adherent erythrocytes was observed after 10 minutes perfusion of ionomycin-treated erythrocytes over histamine-treated ECs. Here, the amount of loosely adherent erythrocytes peaked at 8% (S4 Fig).

These data show that adhesion of erythrocytes to ECs can be induced by calcium accumulation within the erythrocytes and is greatly enhanced upon the activation of ECs by either histamine or thrombin.

### Intracellular calcium accumulation potentiates erythrocyte adhesion to ECs

**Fig 2:**
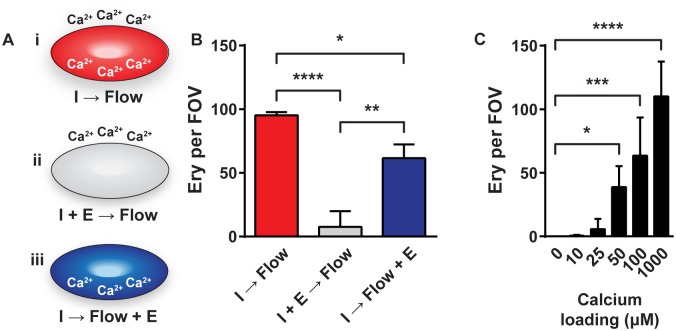
Intracellular calcium accumulation potentiates erythrocyte adhesion to ECs. (A) Schematic representation of the different experimental conditions used to analyze the role of intra- and extracellular calcium for erythrocyte adhesion to ECs. (i) Ionomycin- (1 μM) treated (1 hour) erythrocytes perfused (10 minutes) using a calcium-rich buffer. (ii) Ionomycin plus EGTA- (5 mM) treated erythrocytes perfused using a calcium-rich buffer. (iii) Ionomycin-treated erythrocytes perfused using an EGTA-supplemented buffer. (I = Ionomycin, E = EGTA) (B) Quantification of adhesion of erythrocytes treated as described in panel A perfused (10 minutes) with histamine (100 μM) over HUVECs. (C) Quantification of adhesion of erythrocytes loaded with increasing concentrations of calcium perfused (10 minutes) with histamine (100 μM) over HUVECs. Adherent erythrocytes were quantified as in Fig 1. Values are means ± SD (n = 4; Each n represents single independent experiments using cells from different donors). Statistical analysis was performed using a 1-way ANOVA (B) or a 1-way ANOVA in which all means were compared with 0 μM calcium (C) each followed by Bonferroni's post-hoc test. * *P* < 0.05; ** *P* < 0.01; *** *P* < 0.001; **** *P* < 0.0001.

Before we tested whether ULVWF multimers were involved in the adhesion of calcium-loaded erythrocytes to ECs, we determined the role of calcium in this process. While intracellular calcium is important for cell signaling [23], extracellular calcium is important for maintaining a functional conformation of adhesion molecules [24]. Preventing the ionomycin-induced intracellular increase of calcium (Fig 2Ai) by 5 mM EGTA pre-incubation (Fig 2Aii) caused an almost complete (~92%) reduction of erythrocyte adhesion to histamine-activated ECs (Fig 2B). When extracellular calcium was removed by adding 5 mM EGTA to the flow buffer after the ionomycin-induced calcium influx (Fig 2Aiii), only a 32% reduction in erythrocyte-EC adhesion was observed (Fig 2B). Significantly less erythrocytes adhered to ECs when intracellular calcium was kept low compared with a reduction in extracellular calcium (Fig 2B). When erythrocyte calcium influx was induced in the presence of varying calcium concentrations, increasing erythrocyte-EC adhesion could be observed at a calcium concentration of 25 μM and higher (Fig 2C). A significant increase of erythrocyte-EC adhesion was observed at intracellular calcium levels of 50 μM and higher. (Fig 2C). These results suggest that the adhesion of erythrocytes to activated ECs depends on intracellular calcium accumulation whereas extracellular calcium is not required for erythrocyte-EC binding.

### Von Willebrand factor mediates erythrocyte-EC binding

**Fig 3:**
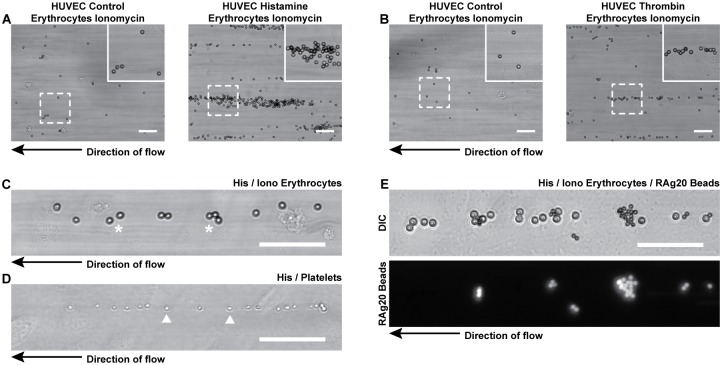
Erythrocytes adherent to activated ECs align in the flow direction. (A) Ionomycin- (1 μM) treated (1 hour) erythrocytes perfused (10 minutes) without (left panel) or with histamine (100 μM) (right panel) over HUVECs. (B) Ionomycin-treated (1 hour) erythrocytes perfused without (left panel) or with (right panel) thrombin (1 U/ml) over HUVECs. The dashed boxes correspond to the zoomed regions. The arrow indicates the direction of the flow. (C) Ionomycin-treated erythrocytes perfused with histamine over HUVECs. Adherent erythrocytes are marked by (*). (D) Platelets perfused (10 minutes) with histamine (100 μM) over HUVECs. Adherent platelets are marked by arrowheads. (E) Ionomycin-treated erythrocytes mixed with monoclonal anti-VWF CLB-RAg20-coupled fluoresbrite YG microspheres (∅ 3 μm) perfused (10 minutes) with histamine over HUVECs. Scale bars represent 50 μm.

Next we determined whether ULVWF multimers from the ECs were involved in the adhesion of calcium-loaded erythrocytes to ECs. Interestingly, calcium-loaded erythrocytes bound at low levels in a random fashion to control-treated ECs (Fig 3A and 3B left panels), while calcium-loaded erythrocytes perfused over histamine- or thrombin-activated ECs adhered in high numbers and clusters of adherent cells aligned in the direction of the flow (Fig 3A and 3B right panels). The alignment of erythrocytes adherent to ECs resembled platelet-binding to VWF (Fig 3C and 3D). We therefore investigated the role of VWF in erythrocyte-EC interaction by perfusing monoclonal anti-VWF coupled beads (CLB-RAg20-beads) together with calcium-loaded erythrocytes over histamine-activated ECs to visualize the VWF strings. The CLB-RAg20-beads bound, together with the erythrocytes, to the ECs and aligned along the same track as the erythrocytes in the direction of the flow (Fig 3E and S5 Fig).

**Fig 4:**
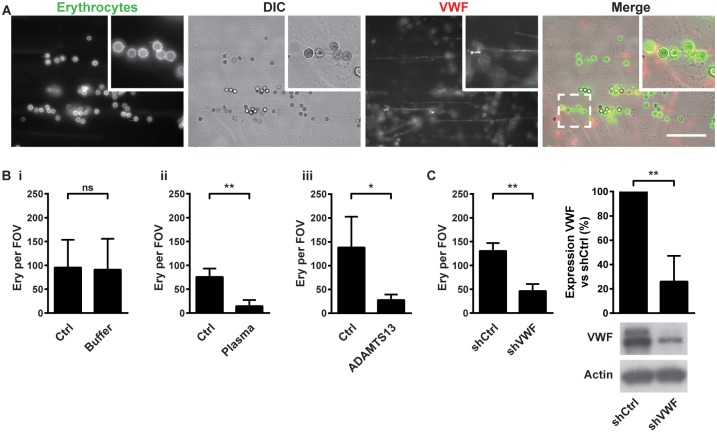
VWF mediates erythrocyte adhesion to ECs. (A) Ionomycin- (1 μM) treated (1 hour) erythrocytes labeled with PKH67 (green), mixed with Alexa Fluor^®^568-labelled anti-VWF antibody (red) perfused (10 minutes) with histamine (100 μM) over HUVECs. The dashed box corresponds to the zoomed region. Scale bar represents 50 μm. (B) Quantification of adhesion of ionomycin-treated erythrocytes perfused (10 minutes) with histamine over HUVECs (Ctrl), followed by 10 minutes perfusion with (i) control buffer (Buffer), (ii) 5% autologous plasma (Plasma), or (iii) 5 μg/ml ADAMTS13 (ADAMTS13). (C) Quantification of adhesion of ionomycin-treated erythrocytes perfused with histamine over HUVECs transduced with lentivirus encoding a control short hairpin (shCtrl) or a short hairpin against VWF (shVWF). Adherent erythrocytes were quantified as in Fig 1. (D) Western blot and densitometric quantification of VWF levels in cell lysates of the lentivirally transduced HUVECs. Values are means ± SD (n = 3; Each n represents single independent experiments using cells from different donors). Statistical analysis was performed using a 2-tailed Student's *t* test. * *P* < 0.05; ** *P* < 0.01.

We also perfused PKH67-labeled calcium-loaded erythrocytes in combination with Alexa Fluor^®^568-conjugated polyclonal anti-VWF antibodies over histamine-activated ECs which confirmed that erythrocytes adhered to EC-derived VWF strings (Fig 4A; S2 Video).

The plasma metalloprotease ADAMTS13 (a disintegrin and metalloproteinase with a thrombospondin type 1 motif, member 13) can cleave VWF strings [25]. The binding of calcium-loaded erythrocytes to histamine-activated ECs did not change after subsequent perfusion of control HEPES buffer (Fig 4Bi), but was significantly reduced after perfusion of 5% autologous plasma or 5 μg/ml recombinant ADAMTS13 over the adherent erythrocytes (Fig 4Bii and 4Biii respectively). This indicates that VWF cleavage prevents erythrocyte adhesion to activated ECs.

Finally, lentivirally transduced short hairpin RNA against VWF significantly reduced the endothelial VWF levels (Fig 4C), caused a loss of VWF-positive endothelial Weibel-Palade bodies (S6 Fig) and reduced VWF string formation upon histamine activation of ECs (S6 Fig). Importantly, VWF knockdown significantly reduced the adhesion of calcium-loaded erythrocytes to histamine-activated ECs (Fig 4C). Together, these results suggest that the adhesion of calcium-loaded erythrocytes to histamine-activated ECs depends on ULVWF strings.

### VWF levels determine binding of calcium-loaded erythrocytes to ECs

**Fig 5:**
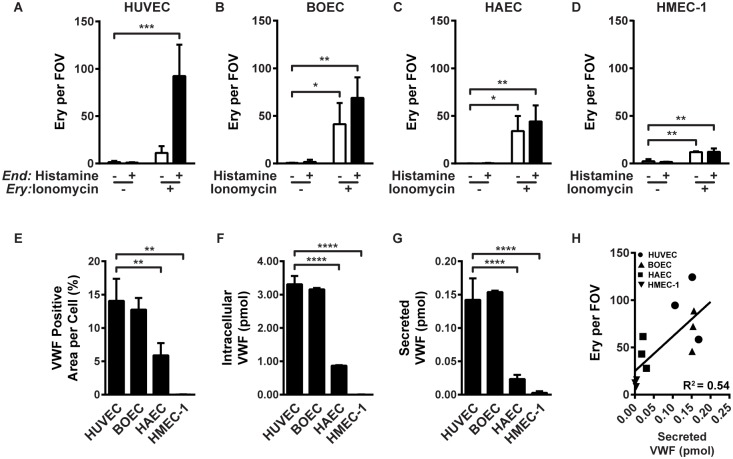
VWF levels determine binding of calcium-loaded erythrocytes to ECs. (A) Quantification of adhesion of ionomycin- (1 μM) or control-treated (1 hour) erythrocytes perfused (10 minutes) with or without histamine (100 μM) over HUVECs, (B) BOECs, (C) HAECs, or (D) the cell line HMEC-1. Adherent erythrocytes were quantified as in Fig 1. (E) Quantification of VWF-positive area per cell based on VE-Cadherin and VWF stained HUVECs, BOECs, HAECs, and the cell line HMEC-1. (F) Intracellular VWF levels and (G) secreted VWF levels after 10 minutes induction by histamine (100 μM) from HUVECs, BOECs, HAECs, and the cell line HMEC-1 measured by ELISA. (H) The absolute number of ionomycin-treated erythrocytes adherent to histamine-treated ECs (obtained from A-D) are plotted against secreted VWF levels (pmol) (obtained from G). Different EC types are marked: ● HUVEC; ▲ BOEC; ■ HAEC; ▼ HMEC1. Values are means ± SD (n = 3; Each n represents single independent experiments using cells from different donors). Statistical analysis was performed using a 2-way ANOVA in which all means were compared with the double-blank control (A-D) or a 1-way ANOVA in which all means were compared with HUVECs (E-G) each followed by Bonferroni's post-hoc test. * *P* < 0.05; ** *P* < 0.01; *** *P* < 0.001; **** *P* < 0.0001.

In addition to HUVECs, we investigated erythrocyte adhesion to blood outgrowth ECs (BOECs), which are more closely related to microvascular endothelial cells [26], human aortic ECs (HAECs), and a human microvascular EC line (HMEC-1). As we observed with HUVECs (Fig 5A), the double hit experimental design induced the most abundant erythrocyte-EC adhesion to BOECs (Fig 5B), HAECs (Fig 5C), and HMEC-1 (Fig 5D), albeit that the absolute number of adherent erythrocytes differed between EC types with lowest binding to the HMEC-1 cell line. Next, we questioned whether differences between intracellular VWF levels of the various EC types could explain the different amounts of adherent erythrocytes. VWF stainings of the different EC types showed a reduction of Weibel-Palade bodies in the HAECs compared to the HUVECs and the BOECs and an almost complete absence in the HMEC-1 cell line (Fig 5E and S7 Fig). This suggests reduced VWF levels in the HAECs and the HMEC-1 cell line. This was confirmed when we measured both intracellular VWF levels and histamine-induced, secreted VWF levels (Fig 5F and 5G). By plotting the absolute number of ionomycin treated erythrocytes adherent to histamine treated ECs against secreted VWF levels a positive correlation between VWF levels and erythrocyte adhesion could be observed (Fig 5H). These data show that erythrocyte-EC adhesion is not specific to HUVECs and can be observed using ECs from different vascular beds, however the extent of erythrocyte adhesion depends on the VWF levels of the ECs.

### Erythrocytes bind independent of platelets to VWF

**Fig 6:**
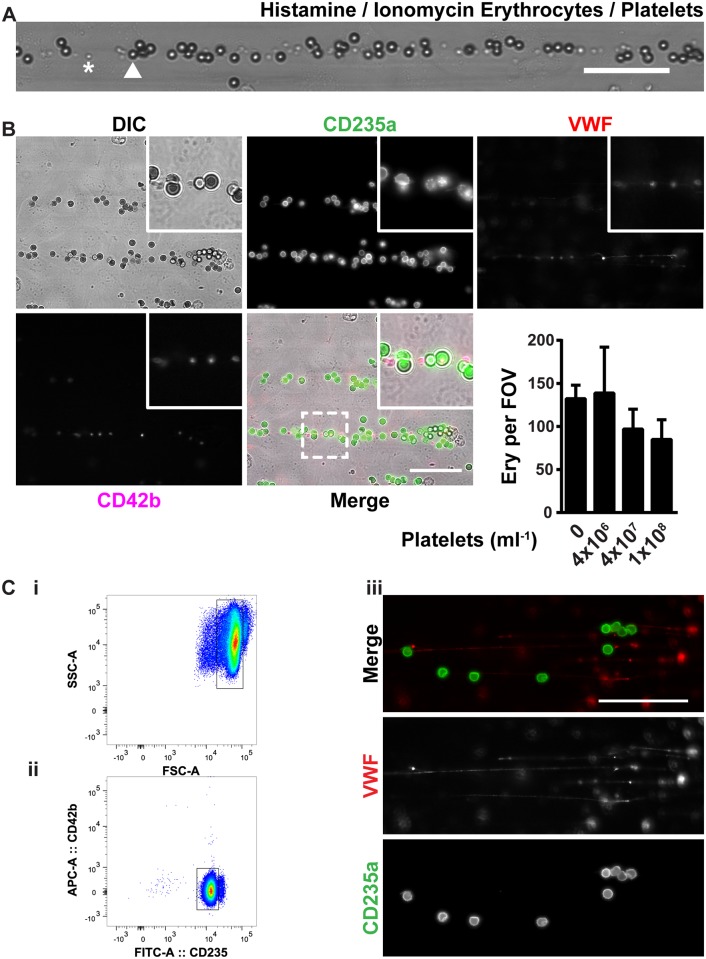
Erythrocytes bind VWF through a platelet-independent mechanism. (A) Ionomycin- (1 μM) treated (1 hour) erythrocytes, mixed with platelets and perfused (10 minutes) with histamine (100 μM) over HUVECs. An example of an adherent platelet is marked by (*) and an adherent erythrocyte is marked by an arrowhead. (B) Ionomycin-treated erythrocytes labeled with anti-CD235a-FITC (green), mixed with anti-CD42b-APC-labeled platelets (magenta) and Alexa Fluor^®^568-labelled anti-VWF antibody (red) were perfused with histamine over HUVECs. Adherent erythrocytes were quantified as in Fig 1. Values are means ± SD (n = 3; Each n represents single independent experiments using cells from different donors). Statistical analysis was performed using a 1-way ANOVA in which all means were compared with 0 platelets ml^-1^ followed by Bonferroni's post-hoc test. (C) Erythrocytes were purified by FACS. The erythrocyte population based on cell size (i) was positive sorted for CD235a-FITC and negative sorted for CD42b-APC (ii). Purified erythrocytes (10^7^ cells/ml) treated (1 hour) with ionomycin (1 μM) (green), mixed with Alexa Fluor^®^568-labelled anti-VWF antibody (red) were perfused with histamine (100 μM) over HUVECs. (iii) The dashed box corresponds to the zoomed region. Scale bars represent 50 μm.

Platelets are the main cells that bind VWF [27]. We questioned whether erythrocytes bind to VWF strings in a fashion independent of platelet binding. Calcium-loaded erythrocytes, mixed with platelets, and perfused over histamine-activated ECs, bound side-by-side to the same VWF strings (Fig 6A and 6B). Furthermore, in the presence of increasing numbers of platelets, there was no significant increase in the number of adherent erythrocytes (Fig 6B).

The platelet integrin α_IIB_β_3_ has been described to bind to the erythrocyte membrane molecule ICAM-4 [28] thereby facilitating erythrocyte binding to VWF [29]. However, we observed platelet-independent binding of erythrocytes to VWF from histamine-activated ECs using live immunofluorescent imaging of anti-CD235a-FITC-labeled calcium-loaded erythrocytes mixed with CD42b-APC-labeled platelets and Alexa Fluor^®^568-labeled anti-VWF antibodies (Fig 6B). Furthermore, erythrocytes purified by FACS, based on the expression of CD235a and lack of CD42b, bound to EC-derived VWF strings (Fig 6C). Together, these results suggest that calcium-loaded erythrocytes can bind, independent of platelets, to VWF strings and that platelets and erythrocytes do not show significant competition for binding sites on ULVWF strings.

## Discussion

**Fig 7:**
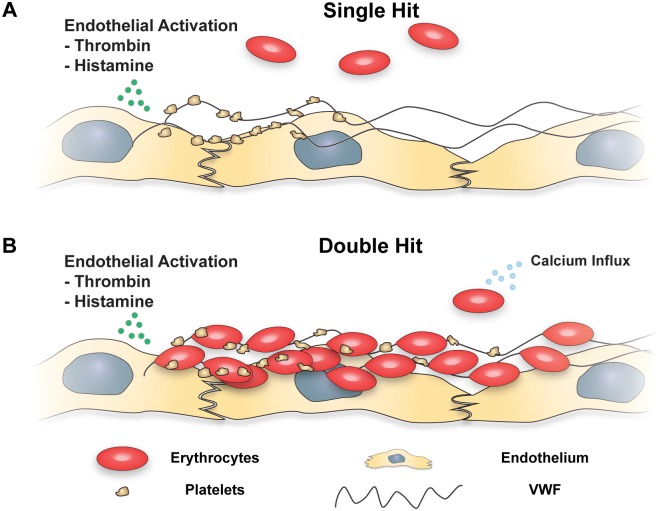
A double hit model describes erythrocyte adhesion to vascular ECs. (A) A single hit model for the adhesion of circulating cells to activated endothelium is shown. Endothelial activation by histamine [22] or thrombin [21] leads to the release of VWF from Weibel-Palade bodies. Platelets adhere to VWF under flow and thereby contribute to primary hemostasis [27]. Normal erythrocytes do not adhere to VWF and remain in circulation. (B) Double hit model for adhesion of circulating cells to ECs. ECs, activated by histamine or thrombin, release VWF from Weibel-Palade bodies. Platelets adhere to the VWF strings. In addition, calcium-loaded erythrocytes can adhere independently of platelets to VWF strings. Micro-thrombi-like structures are formed.

Under physiological flow, erythrocytes do not adhere to the vessel wall. In contrast to erythrocytes, platelets do adhere to the vessel wall under flow, provided the endothelium is activated. Such activation, for example by histamine [22] or thrombin [21], induces the release of VWF from Weibel-Palade bodies. Platelets will adhere to unfolded VWF under flow and thereby participate in primary hemostasis (Fig 7A) [30]. The current study shows that calcium-loaded erythrocytes can also adhere, in a platelet-independent fashion, to VWF strings released by activated ECs (Fig 7B).

Accumulation of calcium within erythrocytes is associated with a number of hereditary anemias including sickle cell disease [23, 31], however calcium influxes into normal erythrocytes have also been described. For instance, calcium influxes were observed after erythrocyte cold storage [32]. Furthermore, the expression of a NMDA receptor channel on erythrocyte membranes has been reported for rat [33] and later in human erythrocytes [34]. It was shown that NMDA receptor agonists including glutamate, *N*-methyl D-aspartate (NMDA), homocysteic acid, and glycine could raise erythrocyte intracellular calcium levels [34]. Furthermore, lysophosphatidic acid (LPA), a second messenger generated by activated platelets that assists in clot formation, was shown to cause a calcium influx into erythrocytes [35, 36]. Whether these stimuli would cause a sufficient raise in intracellular calcium to induce the binding of erythrocytes to VWF remains to be established.

Besides the erythrocyte binding to ULVWF strings which we describe here, erythrocyte adhesion to endothelial cells can also be mediated by many other mechanisms. The endothelial proteins αVβ3 [37], and P-selectin [38] and the erythrocyte proteins BCAM/Lu [2], CD44 [37], and CD36 [39, 40] were also shown to facilitate an EC-erythrocyte interaction. However, if any of these receptors were involved in the adhesion of calcium-loaded erythrocytes to ECs, this interaction should also occur in the absence of ULVWF multimers. Although our results do not rule out a possible partial contribution of adhesion mechanisms, other than through VWF, the ADAMTS13 cleavage- and the VWF-knockdown experiments showed that the majority of calcium-loaded erythrocytes adhere to endothelial cells via ULVWF strings.

The reduced adhesion of calcium-loaded erythrocytes to ECs from microvascular beds (HMEC-1) compared to ECs from macrovascular beds (HUVEC, and HAEC) seems contradictory to previous studies. Microcirculatory studies using *ex vivo* mesocecum vasculature of the rat showed a strong inverse correlation between adhesion of SS erythrocytes and the venular diameter [9, 19]. However, in our experiments we applied a similar wall shear stress on the different EC types, while in the *ex vivo* model, the wall shear stress changes along the vasculature which thereby could interfere with erythrocyte adhesion. Furthermore, we showed a positive correlation between the amount of ULVWF produced by the different types of endothelial cells with the amount of adherent, calcium-loaded erythrocytes. Although the microvascular endothelial cell line HMEC-1 produced barely any VWF, the VWF levels of the primary HUVEC, BOEC, and HAECs were in line with previous results [41]. These differences could explain the apparent contradiction.

Platelets are the main cells to bind to VWF. Erythrocyte ICAM-4 can bind the platelet integrin α_IIB_β_3_ [28]. Thus, platelets could potentially facilitate the erythrocyte-VWF interaction [29]. In this study we show that an ultra-pure erythrocyte population can *in vitro* adhere to VWF in a platelet-independent fashion. This does not exclude that platelet-erythrocyte complexes could enhance erythrocyte-VWF binding, but our data suggests that the contribution of platelets to erythrocyte binding is limited.

The function of erythrocyte binding to VWF is not known. However, increasing evidence shows that erythrocytes play an important role in hemostasis [29]. Their rheological properties cause platelets to be concentrated in the periphery of the blood vessels, close to the vessel wall [42]. In addition, the negative surface of PS-exposing erythrocytes facilitates thrombin generation [43]. Erythrocytes also play an important role in thrombosis. In contrast to white arterial thrombi that consist mainly of platelets, erythrocytes are the major cellular component of red venous thrombi [44, 45]. Our *in vitro* results show that VWF-associated erythrocytes will readily form thrombus-like structures. *In vivo*, VWF^-/-^ mice were shown to be protected from thrombosis in a deep venous thrombosis model by preventing the adhesion of platelets and leukocytes to the vascular endothelium [46]. A similar erythrocyte-VWF interaction may therefore contribute to the formation or propagation of venous thrombi.

In summary, this study shows that erythrocyte adhesion to ECs is rapidly induced when (i) erythrocytes undergo a calcium-influx and (ii) VWF is released from ECs. Our work shows that the adhesion of erythrocytes to ECs is mediated by newly released ULVWF strings and is independent of platelets. The calcium-loaded erythrocytes interact with endothelial-bound ULVWF strings through unidentified receptors. Future work is needed to define the molecular characteristics of erythrocyte receptors that can bind VWF as well as the VWF domain that is responsible for this interaction. Finally, investigating whether the erythrocyte-VWF interactions could play a role in hemostasis and (venous) thrombosis and exploring ways to modulate these interactions will likely prove to be of clinical relevance.

## Supporting information

S1 Fig Example of the standardized method used for quantification of adherent erythrocytes. (A) An example of the raw data acquired which was used for quantification of adhesion of ionomycin-treated erythrocytes perfused with histamine over HUVECs. For each experiment the average number of adherent erythrocytes within 15 fields of view (FOV; 1 FOV = 419.23x319.41 μm) represented by the colored numbers was used. The arrow indicates the direction of flow. The dashed box corresponds to the zoomed region. Scale bar represents 500 μm. (B) Magnification of a single FOV showing the counted adherent erythrocytes marked by turquoise colored numbers (2). Scale bar represents 50 μm. (C) Raw data representing the quantification of adherent erythrocytes from one single condition from one single donor.
(TIF)

S2 Fig Additional controls for erythrocyte and endothelial activation. (A) HUVECs were cultured in perfusion chambers and were stained for VWF (green) and VE-Cadherin (red) or (B) PECAM (red) and nuclei (blue). The dashed boxes correspond to the zoomed regions. Scale bars represent 50 μm. (C-D) Erythrocytes were treated (1 hour) with Ionomycin (1 μM) or DMSO in the presence or absence of EGTA (5 mM). Phosphatidylserine exposure (C) and relative size (D) of Annexin V-FITC and anti-CD235a-PE labeled erythrocytes (10^5^ cells) were determined with a FACSCanto II flow cytometer (BD). (E) Western blot and densitometric quantification of ICAM-1 levels in cell lysates of control- or TNF-α-(10 ng/ml) treated (24 hour) HUVECs. Values are means ± SD (n = 3; Each n represents single independent experiments using cells from different donors). Statistical analysis was performed using a 1-way ANOVA followed by Bonferroni's post-hoc test (C) or a 2-tailed Student's *t* test (D-E). * *P* < 0.05; ** *P* < 0.01; *** *P* < 0.001.
(TIF)

S3 Fig Valinomycin and erythrocyte-EC adhesion. (A) Valinomycin- (10 μM) or control-treated (1 hour) erythrocytes perfused (10 minutes) with or without histamine (100 μM) over HUVECs. Adherent erythrocytes were quantified as in Fig 1. Values are means ± SD (n = 3; Each n represents single independent experiments using cells from different donors). Statistical analysis was performed using a 2-way ANOVA in which all means were compared with the double-blanc control followed by Bonferroni's post-hoc test. (B) Extracellular potassium levels of valinomycin- or control-treated erythrocytes measured by a blood gas analyzer. Values are means ± SD (n = 3; Each n represents single independent experiments using cells from different donors). Statistical analysis was performed using a 1-way ANOVA in which all means were compared with the DMSO control. ** *P* < 0.01; *** *P* < 0.001.
(TIF)

S4 Fig Firmly adherent erythrocytes versus loosely adherent erythrocytes. (A) Absolute number of firmly and loosely adherent erythrocytes after perfusion (2.5, 5, or 10 minutes) of ionomycin- (1 μM) treated (1 hour) erythrocytes with or without histamine (100 μM) over HUVECs. (B) Percentages of firmly and loosely adherent erythrocytes. Erythrocytes were qualified as firmly adherent when they remained immobile for 5 seconds before and 5 seconds after each time point, otherwise they were qualified as loosely adherent. Adherent erythrocytes were quantified as in Fig 1. All bars represent the average of three independent experiments. (n = 3; Each n represents single independent experiments using cells from different donors) The open bars represent firmly adherent erythrocytes. The filled bars represent loosely adherent erythrocytes.
(TIF)

S5 Fig Erythrocytes and CLB-RAg20-beads adhere to ECs. Ionomycin- (1 μM) treated (1 hour) erythrocytes mixed with monoclonal anti-VWF CLB-RAg20-coupled fluoresbrite YG microspheres (∅ 3 μm) (green) perfused (10 minutes) with histamine (100 μM) over HUVECs. The dashed box corresponds to the zoomed region. Scale bars represent 50 μm.
(TIF)

S6 Fig VWF knockdown in ECs. (A) HUVECs lentivirally transduced with a control short hairpin (shCtrl) or a short hairpin against VWF (shVWF) were fixed using 4% PFA and stained for VWF (green) and VE-Cadherin (red). (B) Alexa Fluor^®^568-labelled anti-VWF antibody (red) perfused (10 minutes) with histamine (100 μM) over lentivirally transduced HUVECs expressing a control short hairpin (shCtrl) or a short hairpin against VWF (shVWF). The dashed box corresponds to the zoomed region. Scale bars represent 50 μm.
(TIF)

S7 Fig Weibel-Palade bodies in different types of ECs. HUVECs, BOECs, HAECs, and the cell line HMEC-1 were fixed using 4% PFA and stained for VWF (green) and VE-Cadherin (red). The dashed box corresponds to the zoomed region. Scale bars represent 50 μm.
(TIF)

S8 Fig Individual data points from Figs 1–4. The individual data points from respectively (A) Fig 1, (B) Fig 2, and (C) Fig 4 are shown.
(TIF)

S9 Fig Individual data points from Fig 5 –S4 Fig. The individual data points from respectively (A) Fig 5, (B) Fig 6, (C) S2 Fig, (D) S3 Fig, and (E) S4 Fig are shown.
(TIF)

S10 Fig Original western blot data. (A) The original full scale film of the ICAM-1 control from S2 Fig. (B) The original full scale film of the VWF knockdown check from Fig 4. The first layer of a stacked exposure is shown. (C) The third film layer of the stacked exposure of the VWF knockdown check from Fig 4 is shown.
(TIF)

S1 Video Erythrocyte adhesion to endothelial cells under flow. Ionomycin- (1 μM) or control-treated (1 hour) erythrocytes perfused (10 minutes) with or without histamine (100 μM) over HUVECs.
(AVI)

S2 Video Fluorescently labeled erythrocytes adhere to immunofluorescently labeled VWF under flow. (AVI)
