# Correction: Platelet-independent adhesion of calcium-loaded erythrocytes to von Willebrand factor

**DOI:** 10.1371/journal.pone.0194958

**Published:** 2018-03-21

**Authors:** Michel W. J. Smeets, Ruben Bierings, Henriet Meems, Frederik P. J. Mul, Dirk Geerts, Alexander P. J. Vlaar, Jan Voorberg, Peter L. Hordijk

The Funding section is incorrect. The correct funding information is as follows: R. Bierings and J. Voorberg were supported by funding from the Dutch Thrombosis Foundation.
