# Supplementary figures and images for: Platelet-independent adhesion of calcium-loaded erythrocytes to von Willebrand factor
Source: PLoS One. 2017 Mar 1;12(3):e0173077. doi: 10.1371/journal.pone.0173077 (PMC5332109; doi:10.1371/journal.pone.0173077)

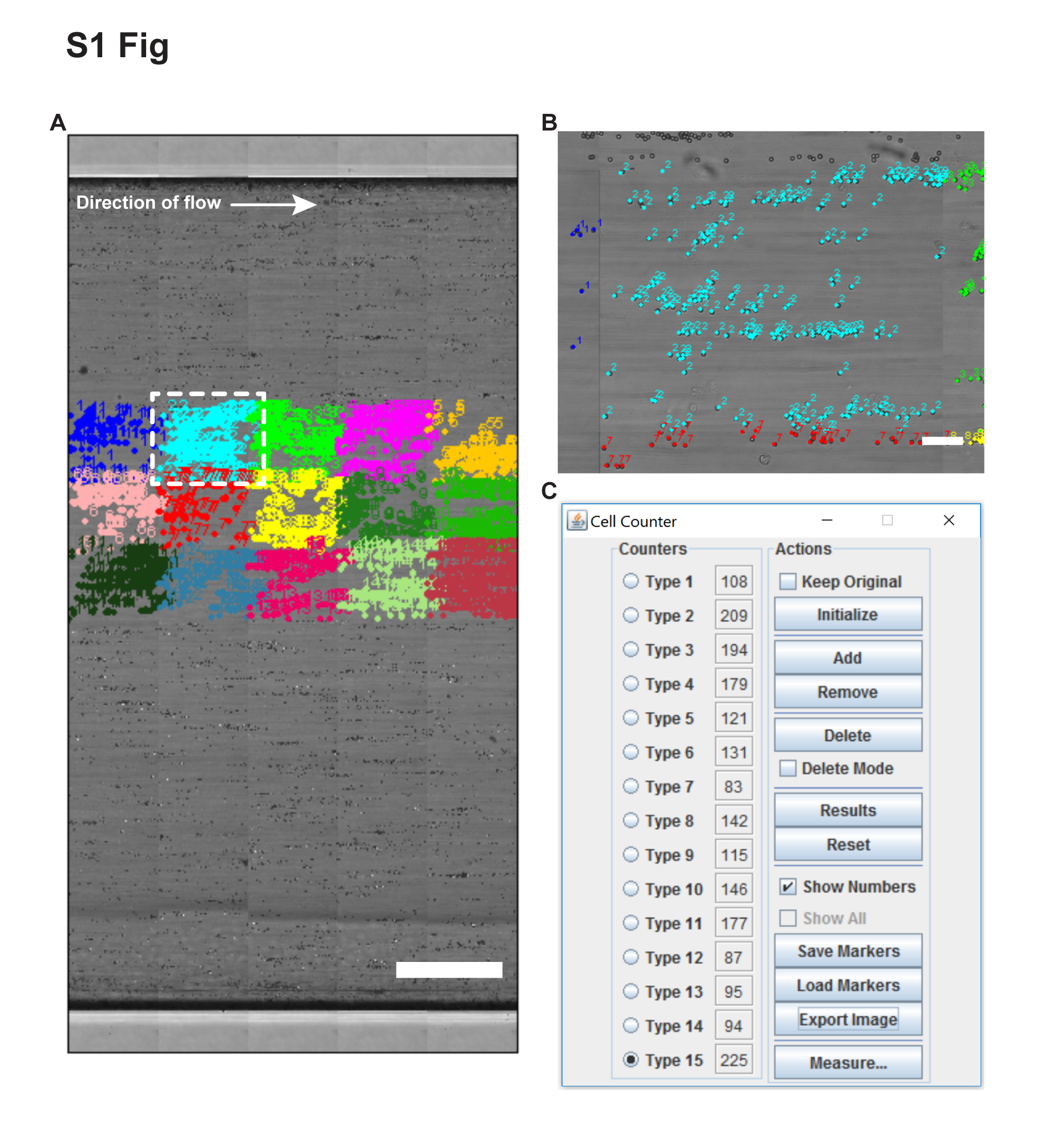

Supplement: S1 Fig — (A) An example of the raw data acquired which was used for quantification of adhesion of ionomycin-treated erythrocytes perfused with histamine over HUVECs. For each experiment the average number of adherent erythrocytes within 15 fields of view (FOV; 1 FOV = 419.23x319.41 μm) represented by the colored numbers was used. The arrow indicates the direction of flow. The dashed box corresponds to the zoomed region. Scale bar represents 500 μm. (B) Magnification of a single FOV showing the counted adherent erythrocytes marked by turquoise colored numbers (2). Scale bar represents 50 μm. (C) Raw data representing the quantification of adherent erythrocytes from one single condition from one single donor. (TIF) [file pone.0173077.s001.tif]

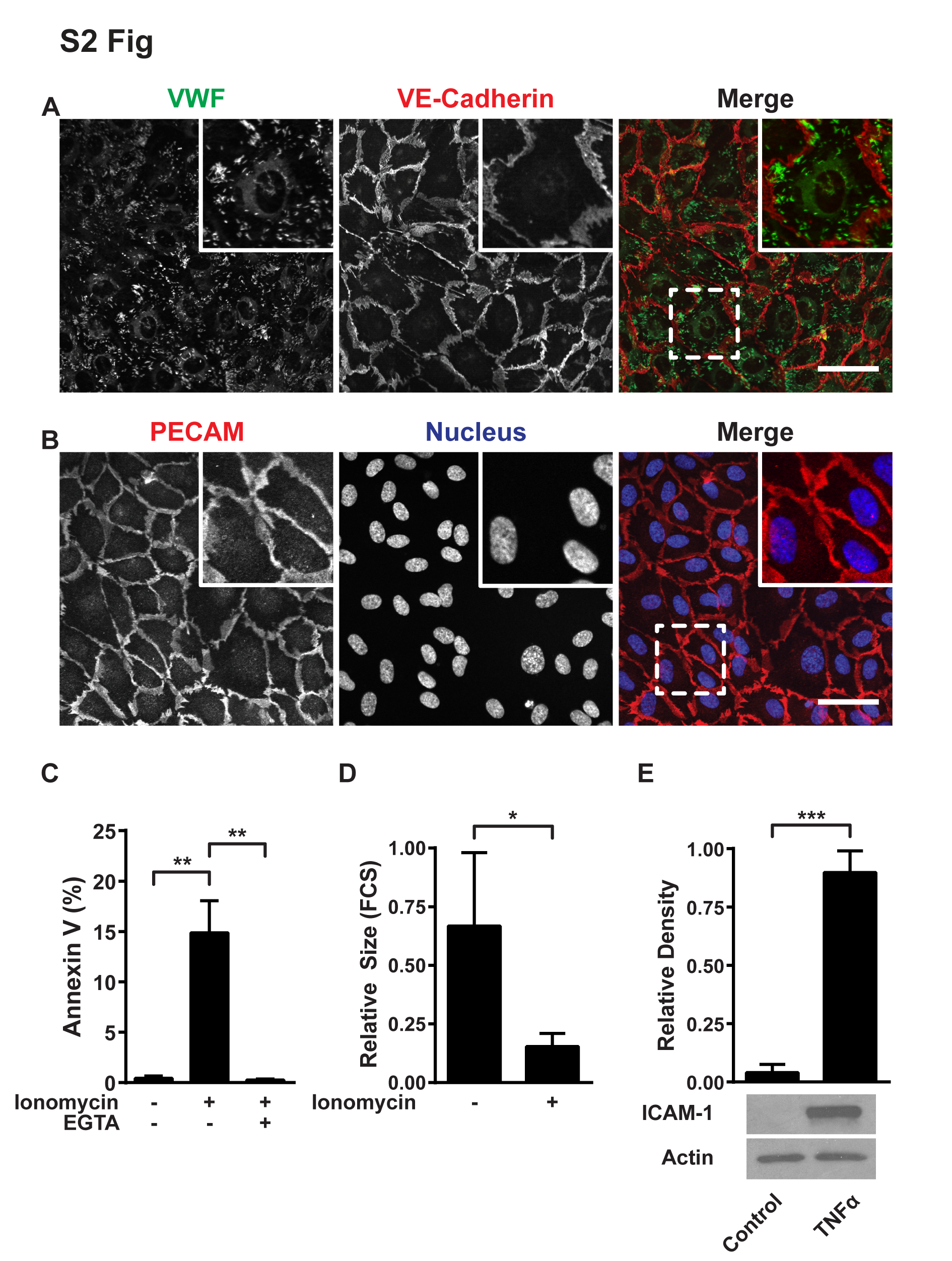

Supplement: S2 Fig — (A) HUVECs were cultured in perfusion chambers and were stained for VWF (green) and VE-Cadherin (red) or (B) PECAM (red) and nuclei (blue). The dashed boxes correspond to the zoomed regions. Scale bars represent 50 μm. (C-D) Erythrocytes were treated (1 hour) with Ionomycin (1 μM) or DMSO in the presence or absence of EGTA (5 mM). Phosphatidylserine exposure (C) and relative size (D) of Annexin V-FITC and anti-CD235a-PE labeled erythrocytes (105 cells) were determined with a FACSCanto II flow cytometer (BD). (E) Western blot and densitometric quantification of ICAM-1 levels in cell lysates of control- or TNF-α-(10 ng/ml) treated (24 hour) HUVECs. Values are means ± SD (n = 3; Each n represents single independent experiments using cells from different donors). Statistical analysis was performed using a 1-way ANOVA followed by Bonferroni's post-hoc test (C) or a 2-tailed Student's t test (D-E). * P < 0.05; ** P < 0.01; *** P < 0.001. (TIF) [file pone.0173077.s002.tif]

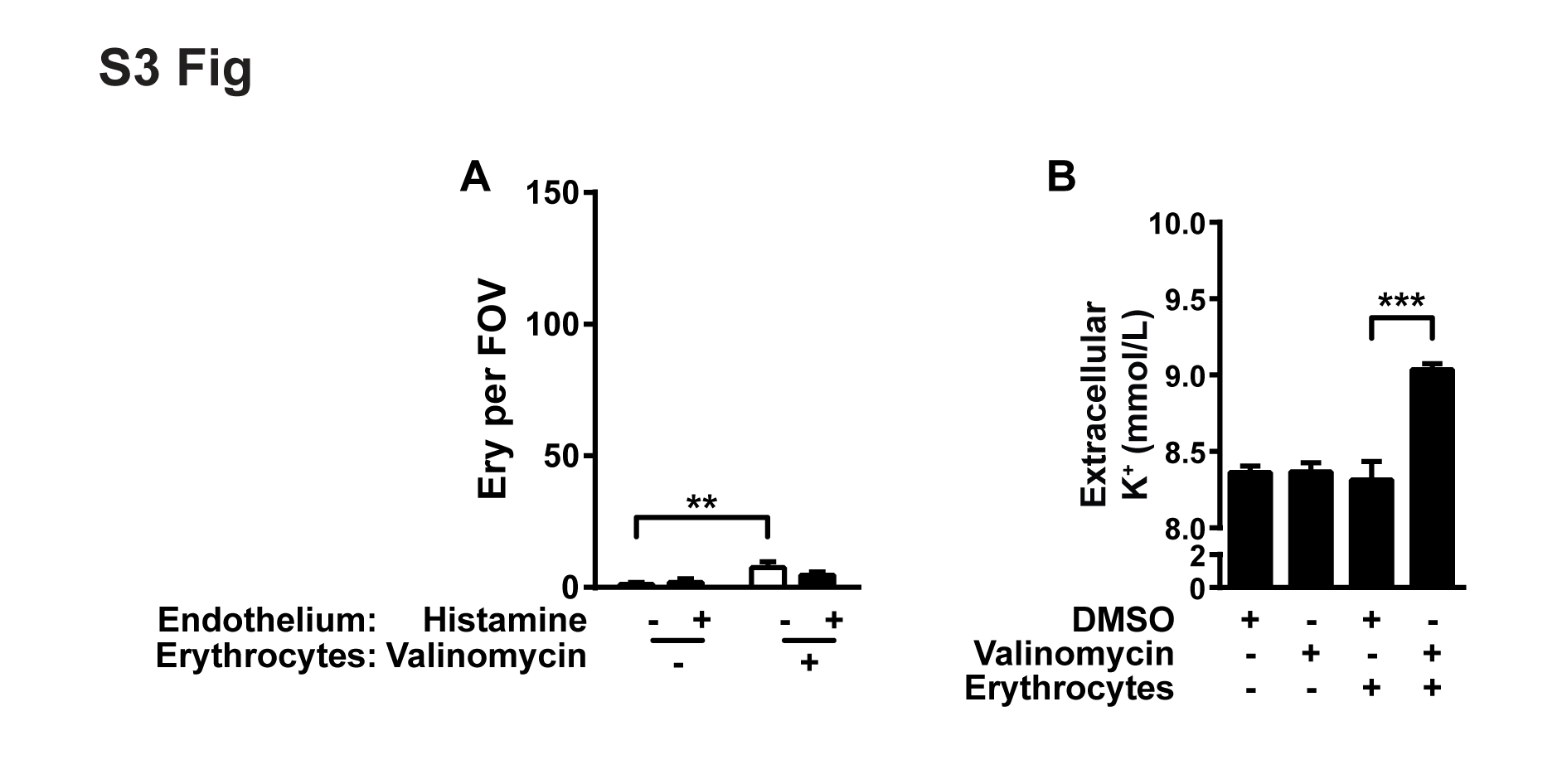

Supplement: S3 Fig — (A) Valinomycin- (10 μM) or control-treated (1 hour) erythrocytes perfused (10 minutes) with or without histamine (100 μM) over HUVECs. Adherent erythrocytes were quantified as in Fig 1. Values are means ± SD (n = 3; Each n represents single independent experiments using cells from different donors). Statistical analysis was performed using a 2-way ANOVA in which all means were compared with the double-blanc control followed by Bonferroni's post-hoc test. (B) Extracellular potassium levels of valinomycin- or control-treated erythrocytes measured by a blood gas analyzer. Values are means ± SD (n = 3; Each n represents single independent experiments using cells from different donors). Statistical analysis was performed using a 1-way ANOVA in which all means were compared with the DMSO control. ** P < 0.01; *** P < 0.001. (TIF) [file pone.0173077.s003.tif]

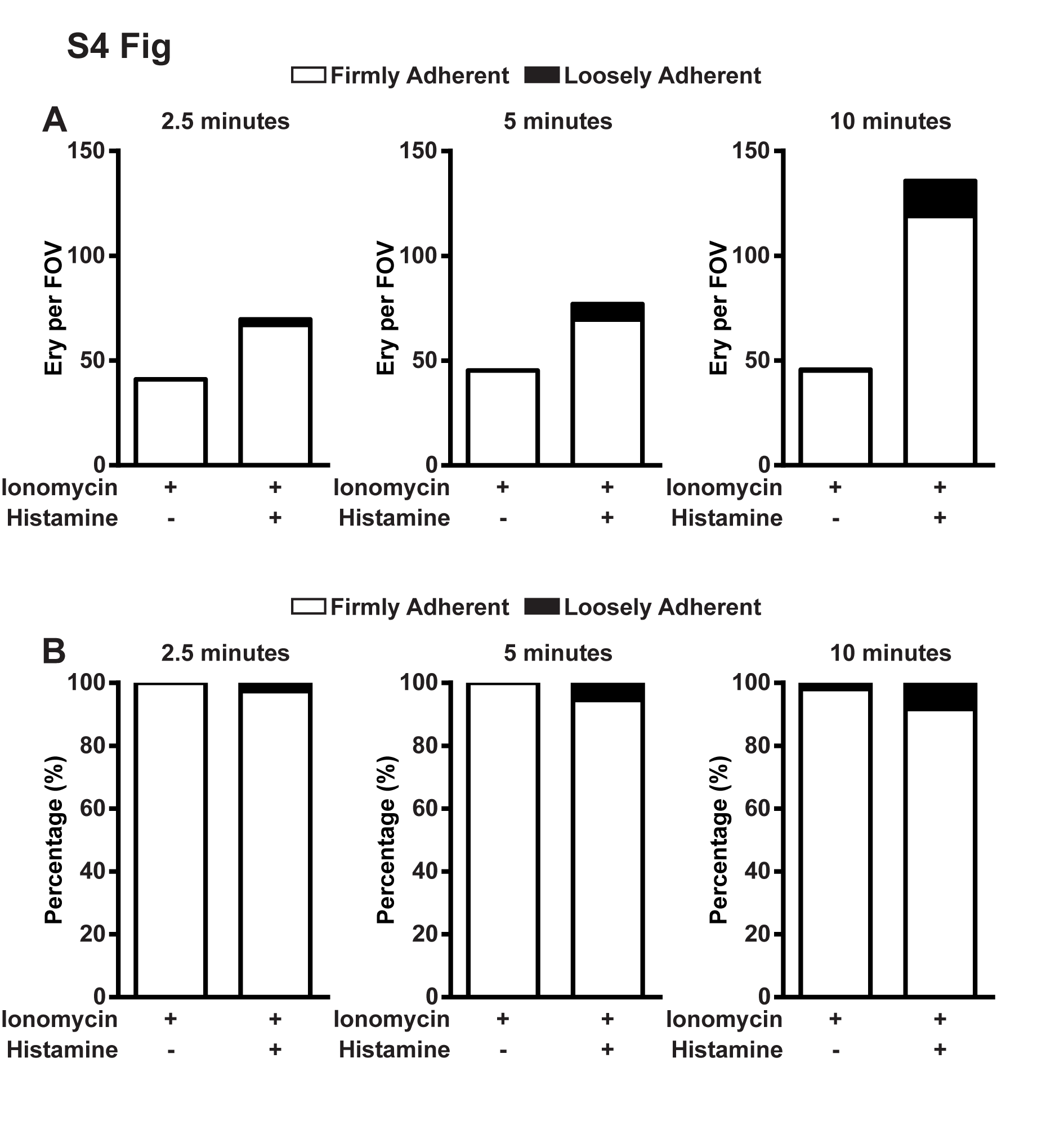

Supplement: S4 Fig — (A) Absolute number of firmly and loosely adherent erythrocytes after perfusion (2.5, 5, or 10 minutes) of ionomycin- (1 μM) treated (1 hour) erythrocytes with or without histamine (100 μM) over HUVECs. (B) Percentages of firmly and loosely adherent erythrocytes. Erythrocytes were qualified as firmly adherent when they remained immobile for 5 seconds before and 5 seconds after each time point, otherwise they were qualified as loosely adherent. Adherent erythrocytes were quantified as in Fig 1. All bars represent the average of three independent experiments. (n = 3; Each n represents single independent experiments using cells from different donors) The open bars represent firmly adherent erythrocytes. The filled bars represent loosely adherent erythrocytes. (TIF) [file pone.0173077.s004.tif]

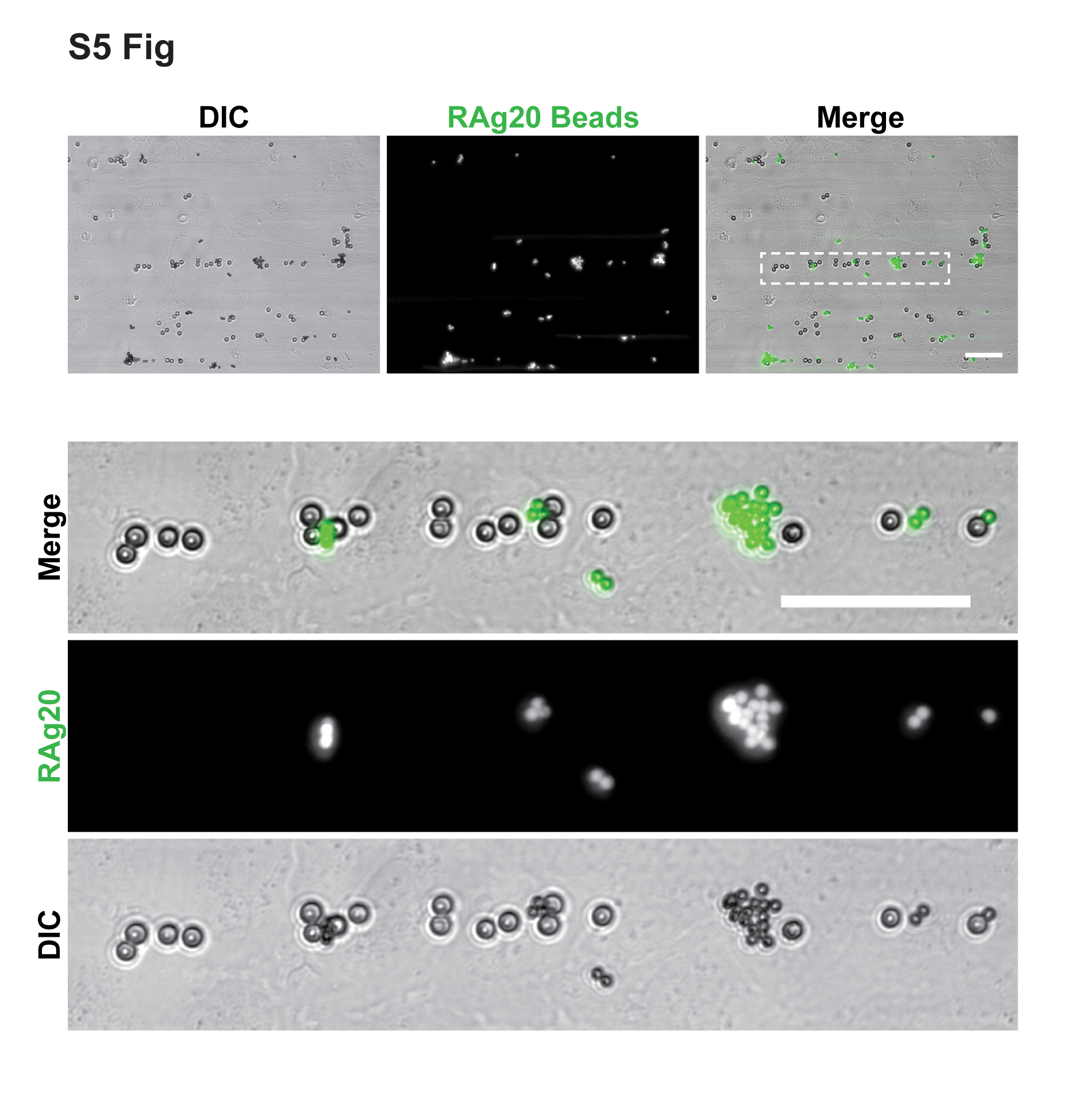

Supplement: S5 Fig — Ionomycin- (1 μM) treated (1 hour) erythrocytes mixed with monoclonal anti-VWF CLB-RAg20-coupled fluoresbrite YG microspheres (∅ 3 μm) (green) perfused (10 minutes) with histamine (100 μM) over HUVECs. The dashed box corresponds to the zoomed region. Scale bars represent 50 μm. (TIF) [file pone.0173077.s005.tif]

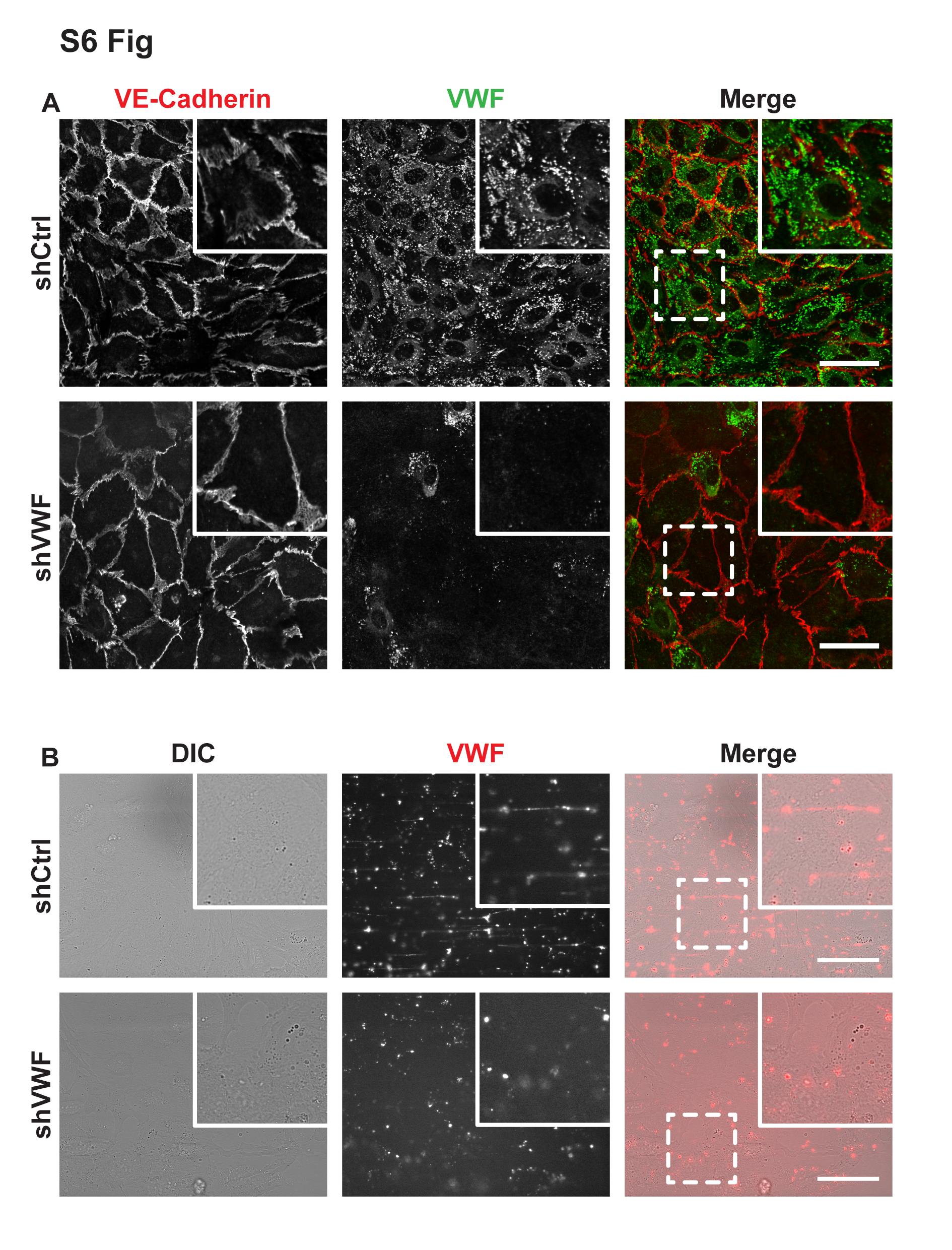

Supplement: S6 Fig — (A) HUVECs lentivirally transduced with a control short hairpin (shCtrl) or a short hairpin against VWF (shVWF) were fixed using 4% PFA and stained for VWF (green) and VE-Cadherin (red). (B) Alexa Fluor®568-labelled anti-VWF antibody (red) perfused (10 minutes) with histamine (100 μM) over lentivirally transduced HUVECs expressing a control short hairpin (shCtrl) or a short hairpin against VWF (shVWF). The dashed box corresponds to the zoomed region. Scale bars represent 50 μm. (TIF) [file pone.0173077.s006.tif]

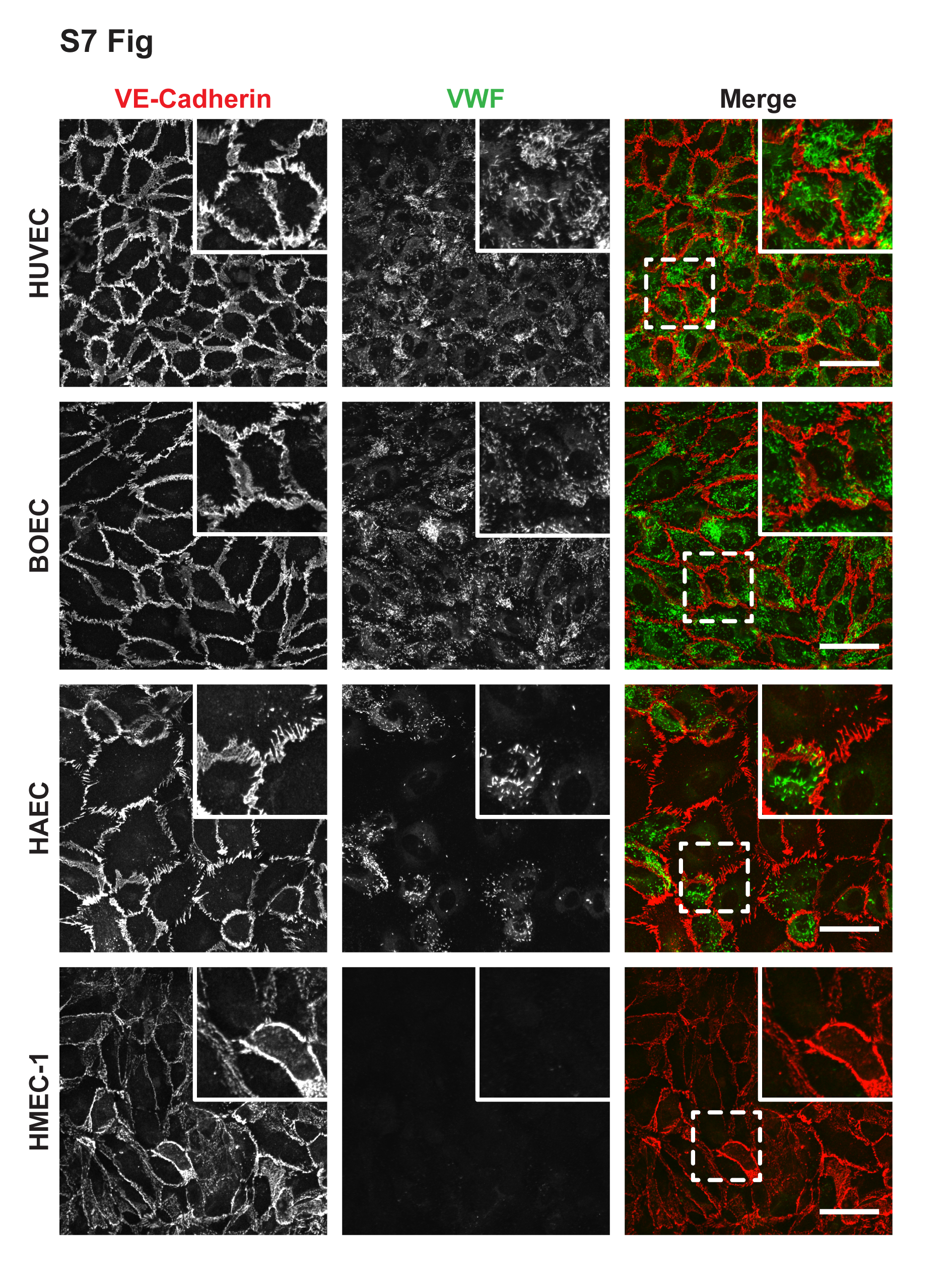

Supplement: S7 Fig — HUVECs, BOECs, HAECs, and the cell line HMEC-1 were fixed using 4% PFA and stained for VWF (green) and VE-Cadherin (red). The dashed box corresponds to the zoomed region. Scale bars represent 50 μm. (TIF) [file pone.0173077.s007.tif]

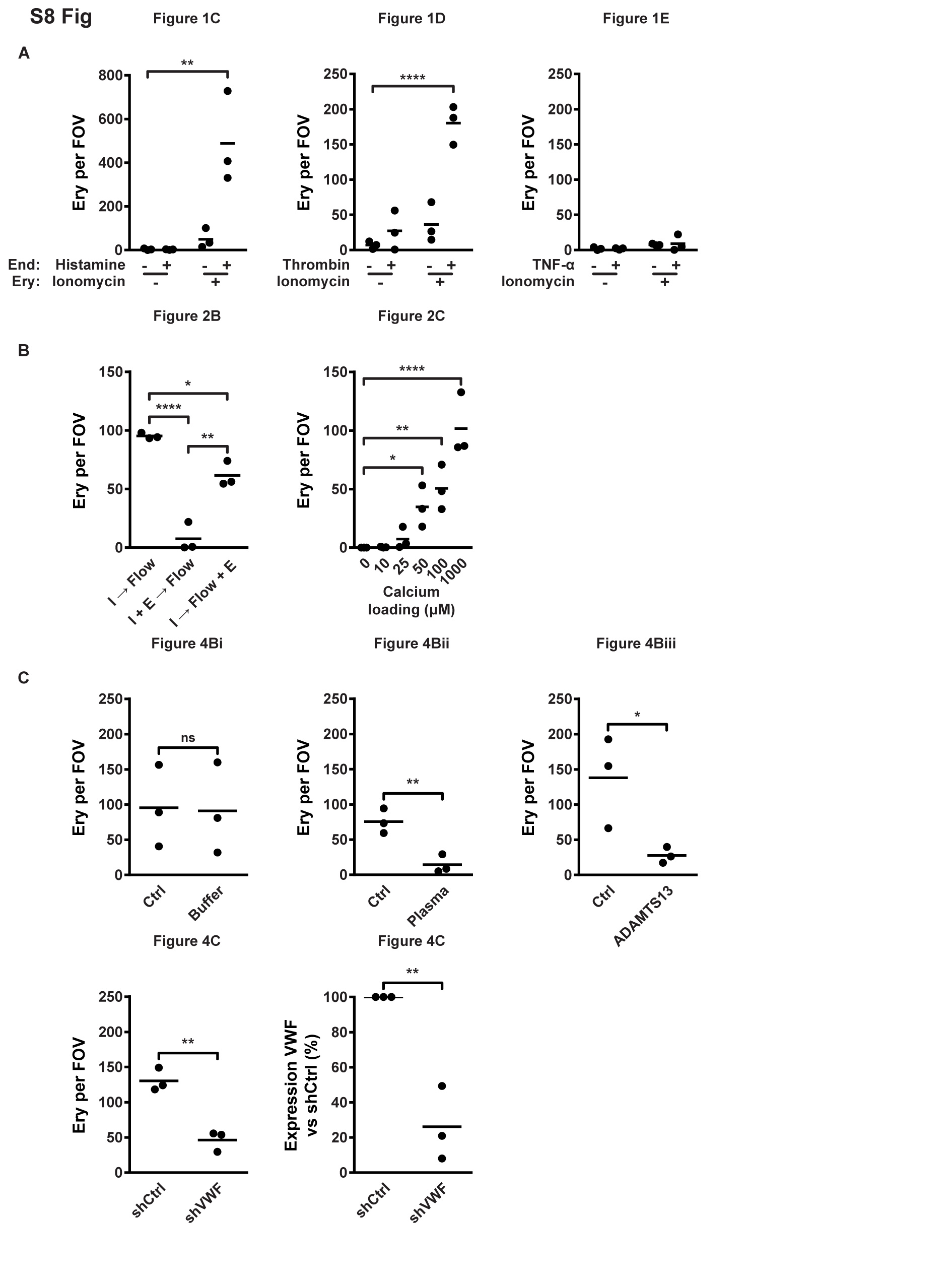

Supplement: S8 Fig — The individual data points from respectively (A) Fig 1, (B) Fig 2, and (C) Fig 4 are shown. (TIF) [file pone.0173077.s008.tif]

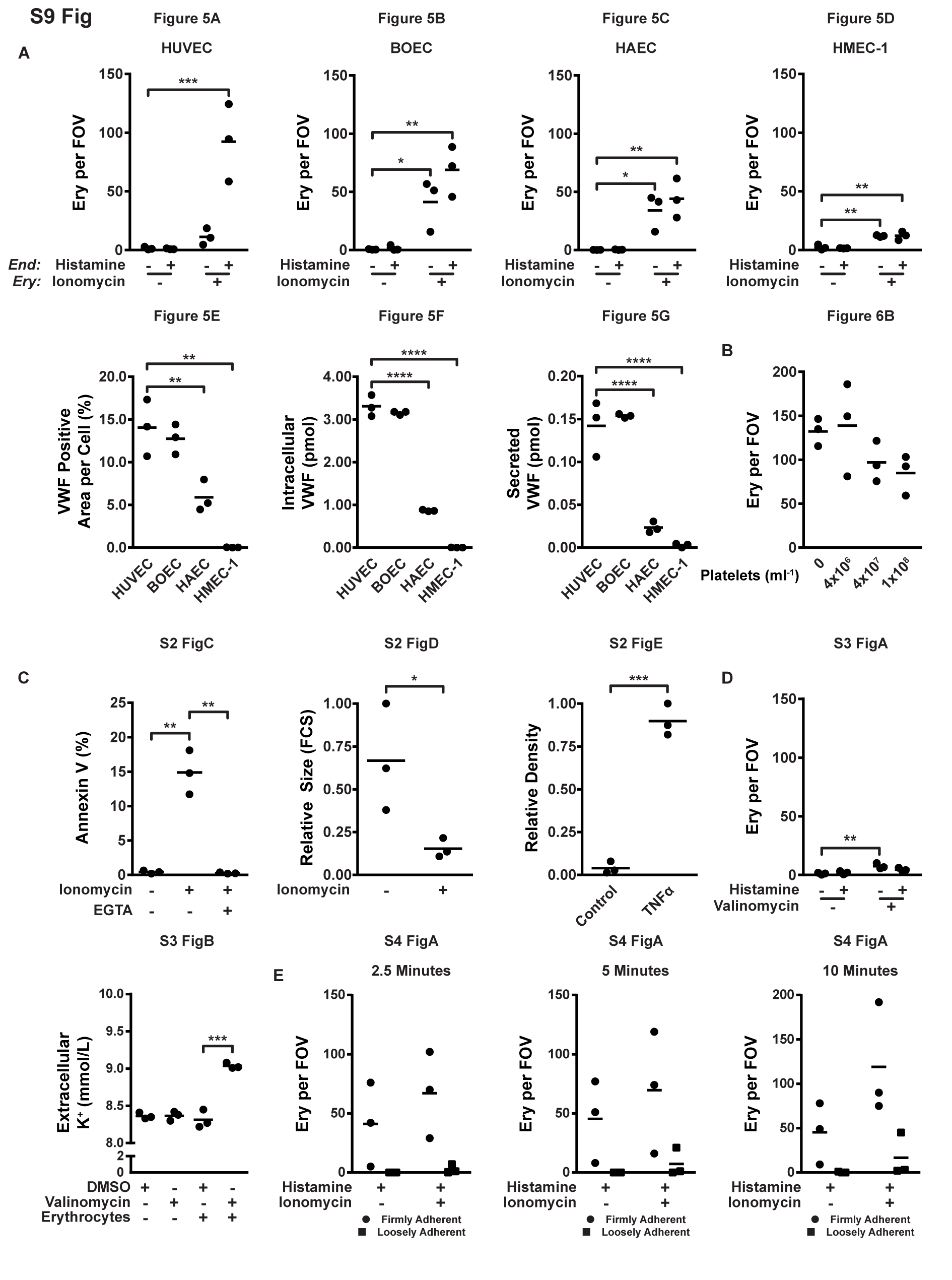

Supplement: S9 Fig — The individual data points from respectively (A) Fig 5, (B) Fig 6, (C) S2 Fig, (D) S3 Fig, and (E) S4 Fig are shown. (TIF) [file pone.0173077.s009.tif]

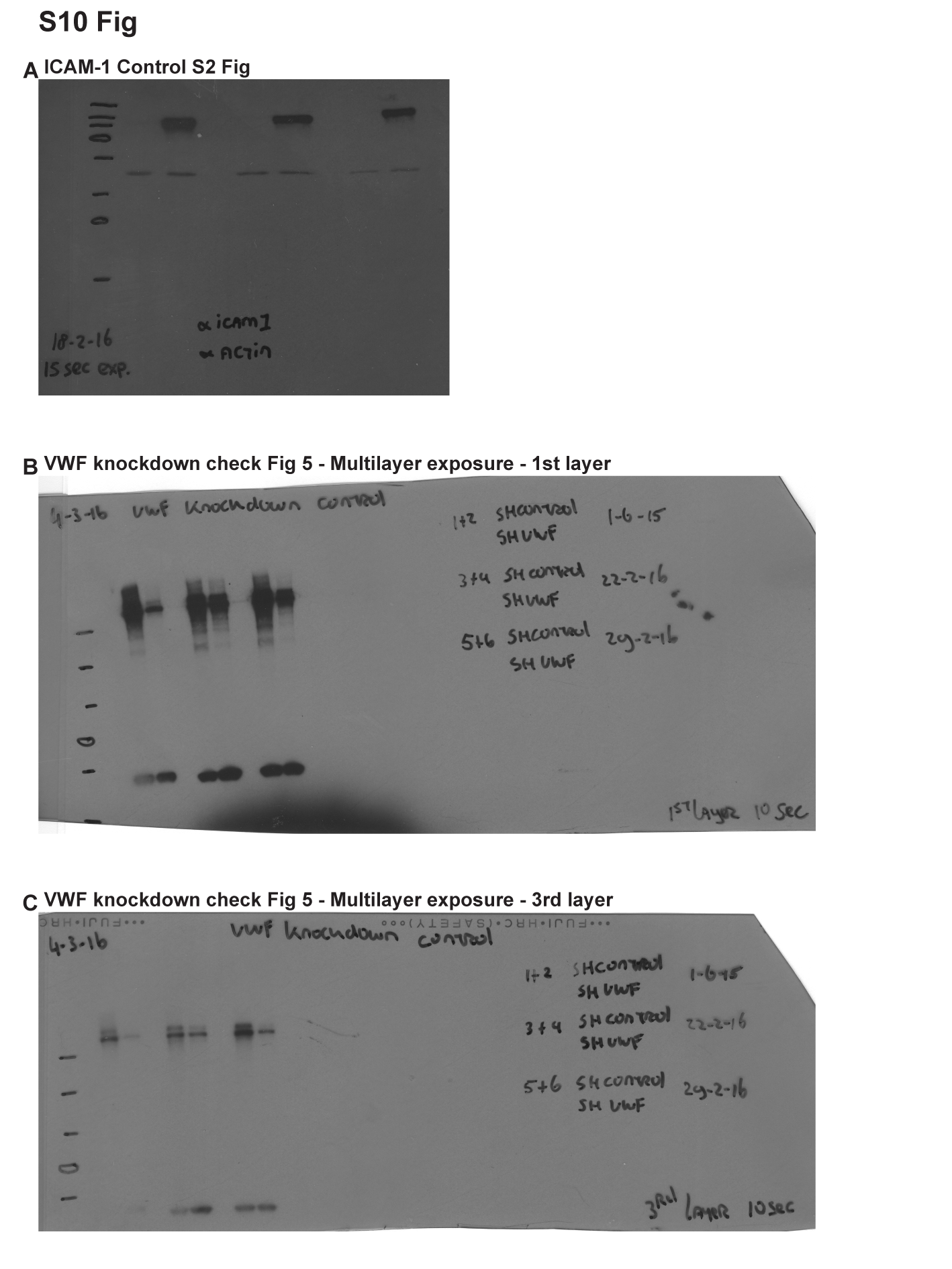

Supplement: S10 Fig — (A) The original full scale film of the ICAM-1 control from S2 Fig. (B) The original full scale film of the VWF knockdown check from Fig 4. The first layer of a stacked exposure is shown. (C) The third film layer of the stacked exposure of the VWF knockdown check from Fig 4 is shown. (TIF) [file pone.0173077.s010.tif]
